# The Importance of Optimal Thermal Ablation Margins in Colorectal Liver Metastases: A Systematic Review and Meta-Analysis of 21 Studies

**DOI:** 10.3390/cancers15245806

**Published:** 2023-12-12

**Authors:** David-Dimitris Chlorogiannis, Vlasios S. Sotirchos, Christos Georgiades, Dimitrios Filippiadis, Ronald S. Arellano, Mithat Gonen, Gregory C. Makris, Tushar Garg, Constantinos T. Sofocleous

**Affiliations:** 1 Department of Radiology, Brigham and Women’s Hospital, Harvard Medical School, Boston, MA 02215, USA; dchlorogiannis@bwh.harvard.edu; 2 Weill-Cornell Medical College, Interventional Oncology/Radiology Service, Memorial Sloan Kettering Cancer Center, New York, NY 10065, USA; 3 Department of Vascular and Interventional Radiology, Johns Hopkins University, Baltimore, MD 21287, USA; 4 2nd Department of Radiology, University General Hospital “Attikon”, Medical School, National and Kapodistrian University of Athens, 12462 Athens, Greece; 5 Division of Interventional Radiology, Department of Radiology, Massachusetts General Hospital, Boston, MA 02114, USA; 6 Department of Epidemiology and Biostatistics, Memorial Sloan Kettering Cancer Center, New York, NY 10065, USA; 7 Department of Vascular and Interventional Radiology, Guy’s and St Thomas Hospital, NHS Foundation Trust, London SE1 9RT, UK

**Keywords:** colorectal cancer, liver metastases, margin, thermal ablation, interventional oncology, systematic review

## Abstract

**Simple Summary:**

Colorectal cancer is a significant cause of cancer-related deaths in the US and the fourth most common malignancy. Percutaneous thermal ablation (TA) is considered a potential alternative to partial hepatectomy in selected patients when eradication of all visible tumor with an ablative margin of greater than 5 mm is achieved. This systematic review and meta-analysis aimed to encapsulate the current clinical evidence concerning the associated risk of different thermal ablation margins for local tumor progression (LTP), while also providing definite evidence concerning the optimal thermal ablation margin. By pooling the available evidence from 21 studies consisting of 2005 participants and 2873 ablated colorectal liver metastases (CLM), this meta-analysis solidifies that a minimal ablation margin over 5 mm is the minimum critical endpoint required, whereas a minimal margin of at least 10 mm yields optimal local tumor control after TA of CLMs.

**Abstract:**

Background: Colorectal cancer (CRC) is the second most common cause of cancer-related deaths in the US. Thermal ablation (TA) can be a comparable alternative to partial hepatectomy for selected cases when eradication of all visible tumor with an ablative margin of greater than 5 mm is achieved. This systematic review and meta-analysis aimed to encapsulate the current clinical evidence concerning the optimal TA margin for local cure in patients with colorectal liver metastases (CLM). Methods: MEDLINE, EMBASE, and the CENTRAL databases were systematically searched from inception until 1 May 2023, in accordance with the PRISMA Guidelines. Measure of effect included the risk ratio (RR) with 95% confidence interval (CI) using the random-effects model. Results: Overall, 21 studies were included, comprising 2005 participants and 2873 ablated CLMs. TA with margins less than 5 mm were associated with a 3.6 times higher risk for LTP (n = 21 studies, RR: 3.60; 95% CI: 2.58–5.03; *p*-value < 0.001). When margins less than 5 mm were additionally confirmed by using 3D software, a 5.1 times higher risk for LTP (n = 4 studies, RR: 5.10; 95% CI: 1.45–17.90; *p*-value < 0.001) was recorded. Moreover, a thermal ablation margin of less than 10 mm but over 5 mm remained significantly associated with 3.64 times higher risk for LTP vs. minimal margin larger than 10 mm (n = 7 studies, RR: 3.64; 95% CI: 1.31–10.10; *p*-value < 0.001). Conclusions: This meta-analysis solidifies that a minimal ablation margin over 5 mm is the minimum critical endpoint required, whereas a minimal margin of at least 10 mm yields optimal local tumor control after TA of CLMs.

## 1. Introduction

Colorectal cancer (CRC) represents the second leading cause of cancer-related mortality in the United States and accounts for the fourth most common malignancy worldwide. Notably, the incidence of CRC among individuals under 55 years has demonstrated a twofold increase from 11% in 1995 to 20% in 2019 [1]. Of the patients who will develop metastatic disease, roughly one-third exhibit hepatic involvement [2,3]. Historically, surgical partial hepatectomy (PH) techniques have been established as the gold standard treatment for selected patients, with a 5-year survival rate between 40 and 60% [4,5]. Nonetheless, 75% of patients with CRC liver metastases are not surgical candidates due to their medical status, advanced disease, and/or comorbidities [3,6]. NCCN recommends Thermal ablation as a standalone therapy or in combination with surgery provided that all visible tumors can be eradicated [7].

Data from large observational trials report that an ablation margin (defined as the shortest distance between the tumor border and the margin of the ablation zone on cross-sectional imaging) of 5 mm is the minimum requirement for acceptable local tumor control [8], while an ablation margin of 10 mm may provide optimal and sustained local tumor control [9,10,11,12]. Despite this, less than 30% of ablated colorectal liver metastasis (CLM) tumors in the published case series reported ablation margins exceeding 10 mm [13,14]. Margin assessment has been historically manually determined via 2D imaging, using the distance between the imaging border of the tumor and the ablation zone as previously described [13]. However, the creation of an ablation zone with MM larger than 5 and especially 10 mm remains challenging. Historically, local tumor progression (LTP) rates ranged between 10 to 52% [15,16], whereas a recent study showed LTP-free survival rates of 74% when margins are 6–10 mm and 80% when margins are over 10 mm [13]. Moreover, emerging evidence suggests that 3D software ablation zone assessment techniques offer improved discrimination value, detecting suboptimal margins and tumor areas at risk for LTP [17,18,19].

This systematic review and meta-analysis is the first that aims to encapsulate the currently available clinical evidence concerning the optimal ablation margin and to correlate oncologic outcomes with post-ablation margin assessments. In addition, it aims to provide definite evidence of 10 mm as the optimal ablation, when thermal ablation is offered as a potentially curative treatment for CLMs.

## 2. Materials and Methods

The design of this meta-analysis followed the PRISMA (Preferred Reporting Items for Systematic Reviews and Meta-Analyses) guidelines and Cochrane Handbook for Systematic Reviews of Interventions [20,21]. The study protocol has been prospectively registered in PROSPERO (CRD42023422713). No ethics board approval was required since no patient was approached, and there was no risk.

### 2.1. Information Sources and Search Strategy

The literature search was conducted by systematically searching Medline (via PubMed), EMBASE (via Scopus), and CENTRAL (Cochrane Central Register of Controlled Trials), as well as the full reference lists of the retrieved studies to identify additional articles (“snowball” method). Databases were searched from inception. A combination of keywords and MeSH (Medical Subject Headings) terms defined the search strategies. The main applied algorithms are available in Supplementary Table S1. The last search date was 1 May 2023, for studies published in English. Two independent investigators conducted the systematic searches, blinded to each other, and any discrepancies were resolved by consensus between them.

### 2.2. Eligibility Criteria

Required inclusion criteria for study are adult patients with metastatic colorectal liver disease treated with thermal ablation for local tumor control of up to three colorectal liver metastases (each less than 5 cm in largest diameter); documented oncologic local tumor progression outcomes of an ablation margin of less than 5 mm vs. equal or greater than 5 mm or a report stratifying by 0–4.9 mm, 5–9.9 mm, and equal or greater than 10 mm in a cohort of patients. Both prospective and retrospective studies were considered eligible. We excluded case-control, comments, cross-sectional, descriptive, animal, and in vitro studies.

### 2.3. Selection Process

The procedure for selecting studies involved three consecutive stages. First, the titles and abstracts of all electronic records were reviewed to identify potential suitability. Second, articles deemed potentially suitable for inclusion were obtained in full-text format. Subsequently, studies that did not meet the inclusion criteria were rejected. Two researchers carried out the selection of studies independently, resolving any discrepancies through an agreement among a third author.

### 2.4. Data Extraction and Data Items

Pre-piloted forms were used for extraction of the following data from the included studies: year of publication, country, study period, design, total sample size, total tumors treated, participants’ median age and sex, mean tumor size, positive lymph node status at the time of disease, mean duration of follow up, *KRAS* mutation status, and post-ablation margin assessment method. Information regarding local tumor progression was also collected. Two independent authors collected the data, resolving any potential discrepancies after a discussion with a third author.

### 2.5. Statistical Analysis

Data analysis was carried out using R-2023.03.1 + 446 (utilizing the “meta” package) [22]. Statistical significance was determined via a two-sided *p*-value threshold of 0.05. Continuous variables were summarized through means and standard deviations, while categorical variables were summarized with absolute values and relative frequencies. In instances where medians and ranges were provided, the approach by Wan et al. [23] was utilized to estimate means and SDs for continuous variables; likewise, when median and interquartile ranges were available, their method was applied. The measure of effect used was the risk ratio (RR) with corresponding 95% confidence intervals (CIs). A random-effect model (Mantel–Haenzel procedure) [24] was employed for the included trials to estimate the pooled RR. In all cases, inverse variance weights were employed. Heterogeneity was tested using inconsistency test (I^2^) statistics (I^2^ = 100% × (Q − df)/Q, where Q = χ^2^, Cochran’s heterogeneity statistic; and df = degrees of freedom). Interpretatively, I^2^ ≤ 25% indicates low heterogeneity, I^2^ ≤ 50% suggests moderate heterogeneity, and I^2^ > 50% signifies high heterogeneity [25]. The presence of publication bias was evaluated through the visual examination of funnel plots. Additionally, a leave-one-out analysis was conducted by iteratively excluding one study at a time and reiterating the statistical analysis.

## 3. Results

### 3.1. Study Selection

**Figure 1:**
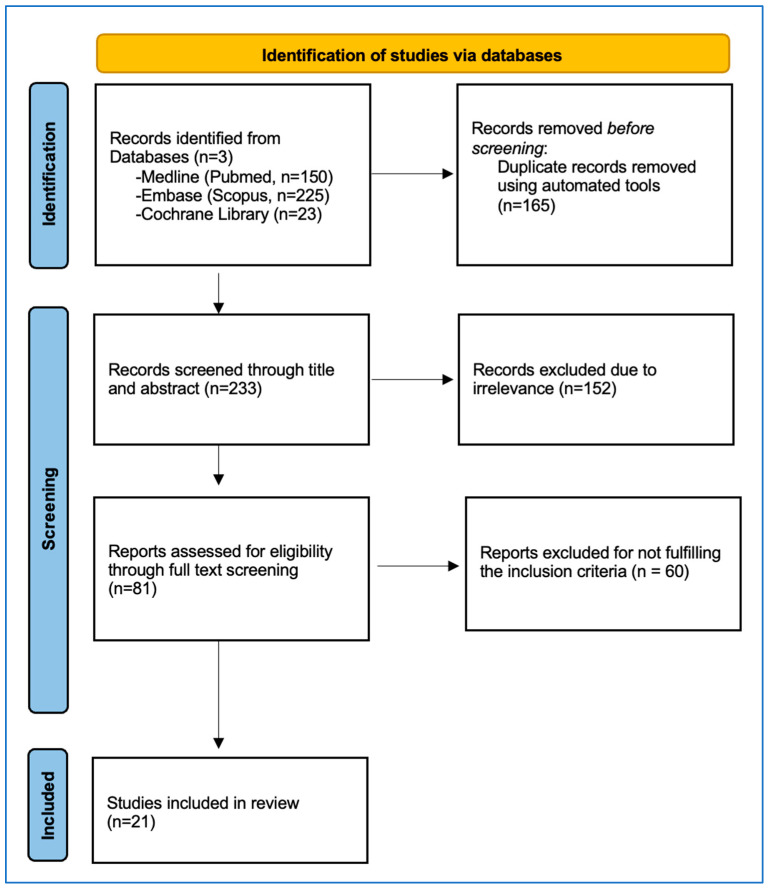
PRISMA Flowchart depicting the detailed study selection process.

The procedure for selecting studies is visually represented in Figure 1 of the PRISMA flowchart. An initial literature search yielded 398 records. After eliminating duplicates, 233 articles were assessed for eligibility, and 81 were retrieved. Following this, 60 studies were excluded because they failed to meet the predefined inclusion criteria. Consequently, 21 studies, amounting to 2005 patients and 2873 ablated CLM, were incorporated in the meta-analysis (as detailed in Table 1).

### 3.2. Included Studies

**Figure 2:**
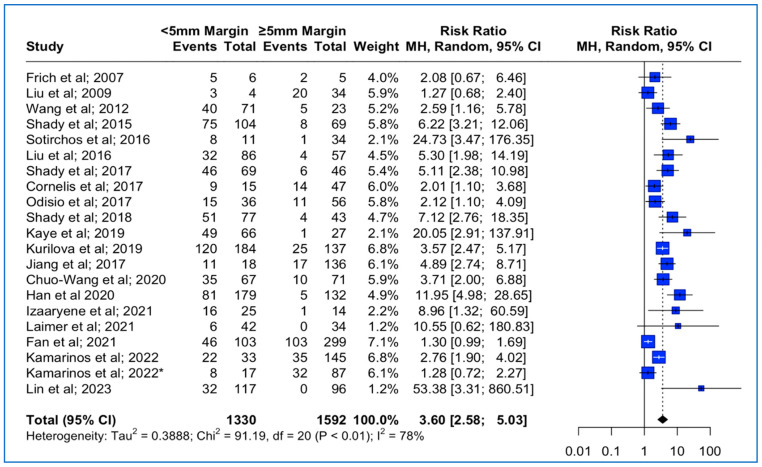
Forest plot of the pooled estimate of the local tumor progression rates between <5 mm vs. ≥5 mm thermal ablation margins (“*”: second study with the same first author and release date).

The methodological characteristics of the included studies are presented in Table 1. Two studies were prospective cohorts, and 19 studies were retrospective cohorts. Among the included studies, 13 out of 21 were from the USA, 4 were from China, 3 were from Europe, and 1 was from South Korea (Table 1). In 18 studies, ablation margin assessment was made using 2D CT imaging (Figure 2). Four studies reported confirmation of the ablation zones and margin-specific local tumor progression outcomes using 3D software techniques [17,18,40,41]. Three studies stratified local tumor progression outcomes by RAS mutation status [31,32,35].

### 3.3. Local Tumor Progression Rates between <5 mm vs. ≥5 mm Thermal Ablation Margin

Data concerning the LTP were extracted from 21 studies (Table 1). The median follow-up time was 23 months (interquantile range (IQR): 18–31). The meta-analysis outcomes regarding the pooled local tumor progression are depicted in Figure 2. The combined data analysis showed that in patients with CLM, treatment with a thermal ablation margin of less than 5 mm was associated with a 3.6 times higher risk for LTP vs. more than 5 mm (RR: 3.60; 95% CI: 2.58–5.03; *p*-value < 0.001). A high heterogeneity between the studies was noted (I^2^: 78%; *p*-value < 0.05). Funnel plot asymmetry evaluation using the Egger’s test revealed significant asymmetry (t = 3.58; *p*-value < 0.002), indicating potential publication bias, while Begg’s test yielded a marginally non-significant result (z = 1.87; *p*-value > 0.06), suggesting weaker evidence against funnel plot symmetry (Funnel plot in Supplementary Figure S1A). Sensitivity analysis using the “leave-one-out” approach did not identify a study that significantly impacted the pooled estimate.

### 3.4. Local Tumor Progression Rates between <5 mm vs. ≥5 mm Thermal Ablation Margins Assessed Using 3D Software Techniques and 2D Methods

**Figure 3:**
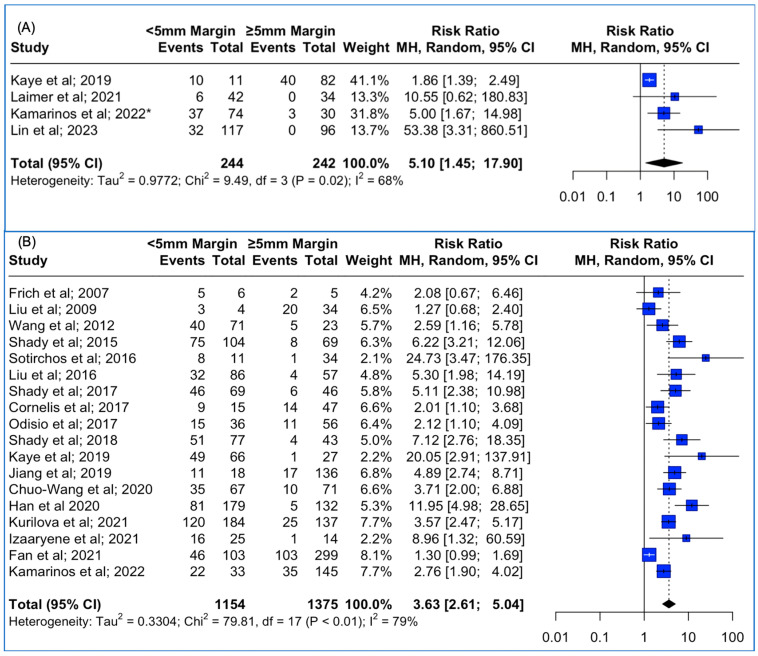
(**A**) Forest plot of the pooled estimate of the local tumor progression rates between <5 mm vs. ≥5 mm thermal ablation margins assessed using 3D software techniques. (**B**) Forest plot of the pooled estimate of the local tumor progression rates between <5 mm vs. ≥5 mm thermal ablation margins assessed using 2D software techniques. (“*”: second study with the same first author and release date).

Data concerning the 3D assessment of the ablation margins were also available from four studies. The meta-analysis outcomes regarding the pooled local tumor progression are depicted in Figure 3A. The combined data analysis showed that in patients with colorectal liver metastases, treatment with a thermal ablation margin confirmed by the usage of 3D software and of less than 5 mm was associated with 5.1 times higher risk for LTP vs. more than 5 mm (RR: 5.10; 95% CI: 1.45–17.90; *p*-value < 0.001). A high heterogeneity between the studies was noted (I^2^: 68%; *p*-value < 0.05). Sensitivity analysis using the “leave-one-out” approach was conducted, meaning that each study was removed and the analysis was repeated without it, allowing the observation of each individual study’s contribution to the overall results. “Leave-one-out” analysis identified that the omission of the study by Kamarinos et al. (2022) [40] resulted in a non-significant pooled estimate (RR 6.91, 95% CI: 0.88–54.4; *p*-value > 0.05).

Data concerning the 2D assessment of the ablation margins were also available from 19 studies. The meta-analysis outcomes regarding the pooled Local Tumor Progression are depicted in Figure 3B. The combined data analysis showed that in patients with colorectal liver metastases, treatment with a thermal ablation margin confirmed by manual 2D assessment and of less than 5 mm was associated with 3.63 times higher risk for LTP vs. more than 5 mm (RR: 3.63; 95% CI: 2.61–5.04; *p*-value < 0.001). The high heterogeneity between the studies was noted (I^2^: 79%; *p*-value < 0.05). Funnel plot asymmetry evaluation using the Egger’s test revealed significant asymmetry (t = 3.46; *p*-value < 0.003), indicating potential publication bias, while Begg’s test yielded a marginally non-significant result (z = 1.63; *p*-value > 0.05), suggesting weaker evidence against funnel plot symmetry. Sensitivity analysis using the “leave-one-out” approach did not identify a study that significantly impacted the pooled estimate.

### 3.5. Local Tumor Progression Rates between <5 mm vs. ≥ 5 mm Thermal Ablation Margins, Based on KRAS Mutation Status

**Figure 4:**
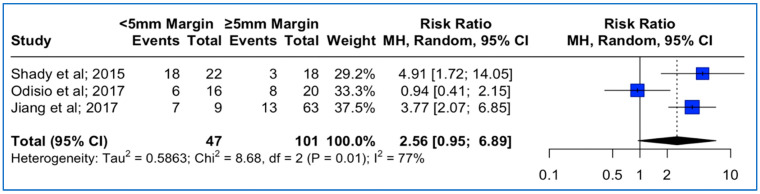
Forest plot of the pooled estimate of the local tumor progression rates between <5 mm vs. ≥5 mm thermal ablation margins assessed in patients with confirmed *KRAS* mutation.

Data concerning ablation margins in tumors with KRAS mutation status were available in three studies. The meta-analysis outcomes regarding the pooled local tumor progression are depicted in Figure 4. The combined data analysis showed that in patients with *KRAS* mutation, CLM treatment with a thermal ablation margin less than 5 mm was associated with a 2.56 times higher risk for LTP vs. more than 5 mm, but this result did not reach statistical significance (RR: 2.56; 95% CI: 0.95–6.89; *p*-value > 0.05). A high heterogeneity between the studies was noted (I^2^: 77%; *p*-value < 0.05).

### 3.6. Local Tumor Progression Rates between <10 mm vs. ≥ 10 mm Thermal Ablation Margin Using Both 3D and 2D Methods

**Figure 5:**
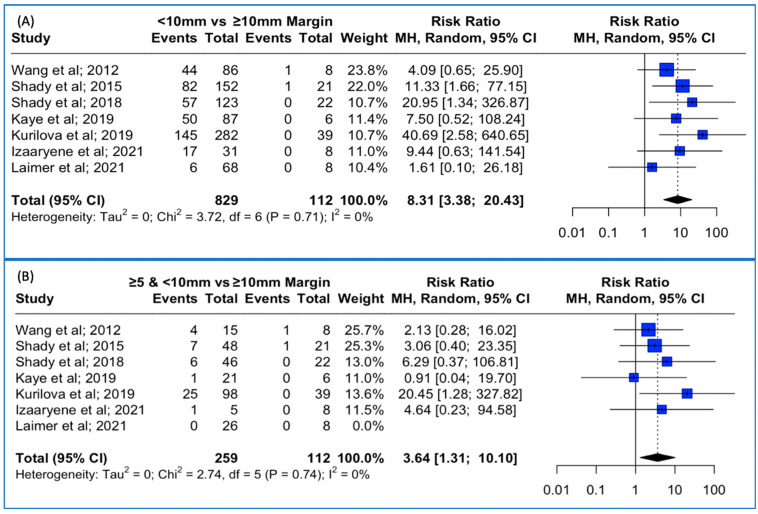
(**A**) Forest plot of the pooled estimate of the local tumor progression rates between <10 mm vs. ≥10 mm thermal ablation margins assessed. (**B**) Forest plot of the pooled estimate of the local tumor progression rates between ≥5 mm and <10 mm vs. ≥10 mm thermal ablation margins assessed.

Data concerning the LTP were extracted from seven studies. The meta-analysis outcomes regarding the pooled local tumor progression are depicted in Figure 5A. The combined data analysis showed that in patients with colorectal liver metastases, treatment with a thermal ablation margin of less than 10 mm was associated with an 8.31-times higher risk for LTP vs. more than 10 mm (RR: 8.31; 95% CI: 3.38–20.43; *p*-value < 0.001). No heterogeneity between the studies was noted (I^2^: 0%; *p*-value > 0.05). Sensitivity analysis using the “leave-one-out” approach did not identify a study that significantly impacted the pooled estimate.

Lastly, when pooling patients with less than 10 mm but over 5 mm ablation margin in the combined analysis, treatment with a thermal ablation margin of less than 10 mm but over 5 mm was associated with a 3.64-times higher risk for LTP vs. more than 10 mm (RR: 3.64; 95% CI: 1.31–10.10; *p*-value < 0.001 (Figure 5B)). No heterogeneity between the studies was noted (I^2^: 0%; *p*-value > 0.05). Sensitivity analysis using the “leave-one-out” approach was conducted, meaning that each study was removed, one at a time, and the analysis was performed without it, allowing us to observe the contribution of each individual study to the overall results. “Leave-one-out” analysis identified that the omission of the study by Kurilova et al. [12] resulted in a non-significant pooled estimate (RR: 2.77, 95% CI: 0.92–8.32; *p*-value > 0.05).

## 4. Discussion

This systematic review and meta-analysis evaluates the pooled local tumor progression rates stratified by ablation margins after thermal ablation for the treatment of colorectal liver metastases. The results of this study suggest that an ablation margin of at least 10 mm is optimal for local tumor control.

Partial hepatectomy has been historically recommended as the “standard of care” for treating metastatic colorectal disease in patients with resectable disease [7,42]. In a meta-analysis of 60 studies by Taylor et al. [43], the median overall survival in patients with CLM who underwent resection was 3.6 years (range: 1.7–7.3 years), and the median overall 10-year survival was 26% (range: 9–69%). Surgical options can be optimized or replaced via the advancement and refinement of minimally invasive techniques, including image-guided percutaneous thermal ablation. While historically, thermal ablation mainly was indicated for patients non-eligible for resection, the current NCCN guidelines also recommend thermal ablation as a standalone therapy or in combination with surgery for selective patients with small tumor burden who can be treated with clear ablation margins, eradicating all visible tumor [7]. A recent meta-analysis that included the results of a randomized phase-II trial underlined the superiority of thermal ablation plus systemic chemotherapy versus systemic chemotherapy alone due to its low complication rates and favorable long-term disease control [44,45]. In the same context, a meta-analysis by Hao et al. reported that radiofrequency ablation offered comparable overall survival rates and disease-free-survival rates to laparoscopic liver resection while also exhibiting lower complication rates (RR: 0.34, 95% CI: 0.230–0.510; *p*-value: <0.05) for the treatment of solitary CLM [46]. Moreover, thermal ablation outcomes for the treatment of CRLM have evolved significantly in terms of efficacy overtime as initially reported by Shady et al. for ablations performed before and after 2009 with the mandatory implementation of contrast-enhanced CT in the immediate assessment of the ablation zone [28] and more recently presented in an analysis of the Amsterdam CORE (AmCORE) registry. In the latter study, Puijk et al. compared the oncologic outcomes between three different periods (2010–2013, 2014–2017, and 2018–2021). The authors highlighted the increasing local tumor progression-free survival over the years for percutaneous ablation; however, they noted that the overall survival of the patients remained stable (5-year survival probability after first thermal ablation: 45.9%) [47]. Using data from the same database, Dijkstra et al. provided long-term thermal ablation outcomes for small-size (0–3 cm) and intermediate-size (3–5 cm) CRLM tumors. The authors underlined that the low incidence of complications, similar overall survival, and relatively high local tumor control rate of 80% may justify thermal ablation for unresectable intermediate-sized CRLM [48]. Even though the literature has matured to a point where a direct comparison between limited hepatic resection and thermal ablation is feasible, there has not been any clear evidence pointing in either direction. A recent systematic review by Kron et al. encompassed the oncologic outcomes of 18 retrospective studies comparing limited hepatic resection and ablation for this matter [49]. The authors identified superior overall survival and disease-free survival of resection compared to ablation for metastatic colorectal disease. However, even though the study presented an extensive overview of the literature, the baseline characteristics of the patients that underwent either treatment were very heterogeneous, with different exclusion and inclusion criteria, and the study did not include any randomized controlled trial, thus limiting the ability to extract significant conclusions. To address the heterogeneity and reporting of oncologic outcomes between studies, an international panel of 62 experts reached consensus on recommendations for the definition of time-to-event endpoints in image-guided thermal ablation. These included assessing per patient, per session, or per tumor outcomes, ending and starting time, survival time definitions, and time-to-event endpoints. Through these guidelines, the authors aimed to reduce subjectivity, avoid results reporting variability in oncological studies, and thus promote effective global communication in the interventional oncology field [50].

Our understanding of outcomes between thermal ablation and resection for small CLM remains limited. The failure and abrupt end of the multicenter (UK and Netherlands) RCT LAVA trial demonstrated the recruitment difficulty due to a possible lack of clinical equipoise among surgeons in the study’s centers and unconscious bias towards surgery [51]. To address the selection bias of the previous retrospective studies, the COLLISION trial is currently ongoing [52]. This phase-III single-blind prospective randomized trial aims to prove non-inferiority for thermal ablation compared to hepatic resection in patients with at least one ablatable and resectable colorectal liver metastasis in the absence of extrahepatic disease. The study is designed to enroll an estimated 618 patients to be registered with an estimated completion date of December 2024. At the Cardiovascular and Interventional Radiological Society of Europe (CIRSE) Congress in 2021 and the European Conference of Interventional Oncology (ECIO) in 2022, its interim results were presented. The investigators confirmed thermal ablation’s excellent safety profile, similar local tumor control, and comparable overall survival rates to partial hepatectomy [53]. Similarly, the HELARC trial (*NCT02886104*) is an ongoing RCT that aims to compare the efficacy of local ablation with hepatectomy for resectable colorectal liver metastatic disease with patients randomized to either surgical resection or microwave ablation. The completion of this study is expected in 2026. In the same context, valuable insights are also expected from another randomized, controlled, multicenter, and non-inferiority trial, the NEW-COMET trial (*NCT05129787*). This RCT has an estimated recruitment population of 230 subjects, and its primary objective consists of the local tumor progression in one year after allocation to either thermal ablation or surgical resection. The estimated completion rate is 2026. Lastly, despite the numerous ongoing trials, the only prospective data to evaluate the non-inferiority of stereotactic microwave ablation to surgical resection stem from the MAVERRIC trial. In this trial, 98 patients who underwent stereotactic microwave ablation were matched to 158 patients from the surgical group. The authors underscored the comparable 5-year overall survival rates after stereotactic microwave ablation, 56% (CI 45–66%) versus 58% (CI 50–66%) after surgery, while the overall and major complications were lower in the stereotactic microwave ablation group (percentage decrease 67% and 80%; *p*-value < 0.01). Thus, this study highlighted the potential of thermal ablation as a valid curative-intent treatment alternative for patients with small colorectal liver metastatic disease [54].

Studies and large case series have identified several independent factors determining the technical success following thermal ablation, with the ablation margin being consistently the most critical factor [9,11,12,13,28,31,33,40,41,54]. A recent panel of experts recommends an ablation margin of 10 mm as the ultimate treatment goal when TA is offered as a local cure for CLM [8]. The results of the current meta-analysis confirm that the risk for LTP is at least 3.6 times higher when the ablation margin is between 5–10 mm versus greater than 10 mm and thus highlights the importance of obtaining a margin of at least 10 mm, while ablation of a CLM with MM under 5 mm is no longer acceptable. Nonetheless, obtaining a margin of at least 10 mm is challenging. While it is strongly recommended in the general patient populations with CLM, it may come at the risk of increased biliary complications in high-risk subgroups of patients. A retrospective analysis of 286 patients with 415 ablated CLMs showed that in a subset of patients previously treated by hepatic arterial chemotherapy (HAC) with floxuridine (FUDR), a minimal ablation margin of >10 mm resulted in a 21% biliary complication rate (*p*-value < 0.001) compared to 0 in the HAC naïve patient group. Additional significant risk factors included prior exposure to bevacizumab and pre-existing biliary dilatation [12]. This reflects the underlying effects of chemotherapy-induced biliary sclerosis (CIBS), a well-known complication of HAC. In this population, combining a minimal ablation margin of at least 5 mm with biopsy-proven complete ablation may provide a safer approach due to the proximity to critical anatomic structures and thus a higher risk for complications. Moreover, in a study by Sotirchos et al. [29], tumor recurrence within the first 12 months after radiofrequency ablation and biopsy-negative ablation zones with minimal margins (≥5 mm) occurred in 1 (3%; 95% CI: 0–9%) of 34 patients. In the same context, in a study by Kamarinos et al. [39], the 12-month rate of LTP for a tumor-negative biopsy ablation zone with margins of at least 5 mm was 7% (95% CI: 3, 14), suggesting this method as a viable treatment option in this population that cannot be ablated with wider margins.

Confirming the achieved ablation margin and the location of the minimal margin is as critical as the margins’ size. A recent study by Schaible et al. pointed out that there is high inter- and intra-reader variability when assessing ablation margins with 2D imaging (intraclass correlation coefficient (ICC): 0.36 (95%CI 0.19–0.52)) [55]. In this context, the evolution of technology has offered a valuable tool in the therapeutic armamentarium [18]. Three-dimensional state-of-the-art software-assisted imaging has recently been proposed as a more accurate method of minimal margin assessment for detecting areas in the tumor ablation zone that have not been adequately covered. In a retrospective study of 68 consecutive patients with 104 CLMs, Kamarinos et al. [40] reported that if the 3D-margin assessment were available intraoperatively, an additional ablation would have been offered in 26/37 (70%) cases to achieve a minimal margin of at least 5 mm. In this systematic review and meta-analysis, four studies included stratification of local tumor progression rates based on ablation margins. Confirmation by using 3D software and of less than 5 mm was significantly associated with a 5.1 times higher risk for LTP vs. more than 5 mm (RR: 5.10; 95% CI: 1.45–17.90; *p*-value < 0.001). Despite these favorable results and the superiority of the 3D imaging assessments, there are several limitations. First, the standard error of the measurements has not clearly been defined and dramatically varies depending on the imaging parameters of the original CT imaging imported for analysis. Second, the software’s ability to accurately account for the post-ablation tissue shrinkage phenomenon, especially after microwave ablation, remains limited. And lastly, the discrimination power of such software is still influenced by patient’s movement, table position, and imaging registration errors that directly affect the accuracy of calculations and resulting measurements. This was apparent in the study by Laimer et al. [18], where receiver operating characteristic (ROC) analysis demonstrated limited precision of the 3D measurements to predict local tumor progression (LTP) when dealing with suboptimal margins between 1–2 mm. Even though the current literature regarding the oncologic outcomes after thermal ablation has matured enough to provide a comparable result to surgical techniques [11], there is still an unmet need for a reproducible technical endpoint for curative intent tumor ablation. The recently opened ACCLAIM clinical trial (Ablation with Confirmation of Colorectal Liver Metastases; ClinicalTrials.gov registration no. *NCT05265169*) aims to address this issue and establish a minimum thermal ablation reproducible endpoint through the 3D minimal margin confirmation. This prospective multicenter international trial by the Society of Interventional Oncology will aim to enroll subjects with one-to-three CLMs with each up to 2.5 cm in largest diameter (for a total of 330 tumors/approximately 275 subjects) eligible for local cure using MWA to validate the impact of a confirmed ablation margin of at least 5 mm on LTP, LTP-free survival, and hepatic-disease-free survival. The estimated completion date is in late 2027.

Another patient-related factor that is correlated with patient survival is the mutation of the *RAS* family gene, with the incidence of such mutations reaching as high as 42.3% in the reported literature [56]. Several studies have identified a decreased overall survival in patients with RAS mutations after systemic chemotherapy, resection, and thermal ablation [9,56,57]. The risk for earlier LTP is fifteen times higher than the wild-type RAS when treated with insufficient margins [31]. In a prior study, it was shown that in RAS mutant CLM, there was no difference in LTP when treating the tumor with margins 6–10 (LTP: 40%) or 1–5 mm (LTP: 0.38%) (RR: 0.94; 95% CI: 0.41–2.15; *p*-value > 0.05), indicating that a margin under 10 mm margin, as depicted by 2D methods, is inefficient for these tumors [28]. This information underscores the need for a larger ablation margin to mitigate LTP risk, especially in the RAS mutant CLM. This result further confirms the findings of a two-institutional retrospective analyses by Calandri et al., where a margin of at least 10 mm significantly impacts the 3-year LTP-free survival rates in this population vs. less than 10 mm margin (48% and 29%, respectively; *p*-value < 0.05) [9]. Lastly, in a subsequent retrospective study by Lin et al. evaluating 213 ablated CLMs in 124 patients using state-of-the-art 3D margin confirmation, *RAS* mutation was no longer an independent risk factor for LTP, suggesting the further efficacy of the 3D software confirmation [14].

This systematic review and meta-analysis is not without limitations. First, the quantitative synthesis was performed using study-level data rather than patient-level data, which could have been employed to adjust for baseline demographic factors that might introduce confounding bias and lead to the depicted high heterogeneity of the studies. Second, concerning the LTP rates, provided the overall thermal ablation margins between <5 mm vs. ≥5 mm are assessed using 3D software techniques, the “leave-one-out” sensitivity analysis identified that the omission of the study by Kamarinos et al. (2022) [40] resulted in a non-significant pooled estimate. Similarly, when pooling patients with less than 10 mm but over 5 mm ablation margin in the combined analysis, the omission of the study by Kurilova et al. [12] also resulted in a non-significant pooled estimate. This finding suggests that the results may be driven by the inclusion of each respective study, possibly raising potential concerns about its contribution to the overall effect size and generalizability of these results. Nonetheless, it is important to note that specific factors likely contribute to this finding, including the different study design characteristics, sample size, or methodological differences, and not the quality of the respective study. In the same context, the studies included had different study durations and patient characteristics and were predominantly observational, with most lacking randomizations and with the comparisons being made without adjusting for the probability of patients receiving treatment based on the respective ablation margin (<5 mm, ≥5 mm, and <10 mm, and >10 mm) further increasing the between-study heterogeneity. Moreover, for the statistical analysis of the LTP rates, the RR was selected as a measure of effect instead of the Hazard Ratio due to the inconsistent reporting between the studies and it being the only method to pool the reported results, making the generalization of these results challenging and lowering the certainty of the evidence. In addition, publication bias could not be assessed reliably as the number of included studies per outcome was less than the required cutoff (*n* = 10). Nevertheless, the overall outcomes of this study mirror a representation of real-world data and are in accordance with the existing funds of knowledge.

## 5. Conclusions

This meta-analysis concludes that a 10 mm minimal thermal ablation margin provides optimal results for CLM, while a 5 mm minimal margin is critical for local tumor control. A margin smaller than 5 mm is no longer to be considered acceptable for local tumor control, especially in patients with KRAS-confirmed mutation. This finding was more prominent in studies that used 3D software for ablation margin confirmation.

## Figures and Tables

**Table 1:** Characteristics of the included studies.

Study Name	Country	Study Design	Total Sample Size	Total Tumors Treated	Sex (Male)	Sex (Female)	Mean Tumor Size (cm)	Follow-Up (Months)	Margin Assessment with 3D Software
Frich et al., 2007 [26]	Norway	Retrospective	11	11	NA	NA	2.2	9	No
Liu et al., 2009 [27]	USA	Retrospective	28	38	24 *	14 *	2.57	19	No
Wang et al., 2012 [13]	USA	Retrospective	73	94	ΝA	ΝA	ΝA	20	No
Shady et al., 2015 [28]	USA	Retrospective	162	233	NA	NA	NA	55	No
Sotirchos et al., 2016 [29]	USA	Prospective	47	67	32	15	2.1	12	No
Liu et al., 2016 [30]	China	Retrospective	101	143	64	37	2.1	21.1	No
Shady et al., 2017 [31]	USA	Retrospective	97	113	NA	NA	NA	60	No
Odisio et al., 2017 [32]	USA	Retrospective	92	137	62	30	NA	12	No
Shady et al., 2018 [33]	USA	Retrospective	110	145	73	37	1.75	NA	No
Cornelis et al., 2017 [34]	USA	Retrospective	39	62	19	20	NA	22.5	No
Kaye et al., 2019 [17]	USA	Retrospective	72	93	44	28	1.8	24	Yes
Jiang et al., 2019 [35]	China	Retrospective	76	152	49	27	NA	32	No
Zhuo Wang et al., 2020 [36]	China	Retrospective	85	138	56	29	2.8	30	No
Han et al., 2020 [11]	South Korea	Retrospective	221	311	155	66	NA	43	No
Kurilova et al., 2021 [12]	USA	Retrospective	286	415	NA	NA	NA	31	No
Izaaryene et al., 2021 [37]	France	Retrospective	84	84	32	7	NA	13.3	No
Laimer et al., 2021 [18]	Austria	Retrospective	45	76	31	14	2.4	36.1	Yes
Fan et al., 2021 [38]	China	Retrospective	199	402	124	75	1.6	23	No
Kamarinos et al., 2022 [39]	USA	Prospective	107	182	65	42	2	31	No
Kamarinos et al., 2022 [40]	USA	Retrospective	68	104	23	45	1.9	21	Yes
Lin et al., 2023 [41]	USA	Retrospective	124	213	55	69	1.4	24	Yes

* Based on tumors treated.

## Supplementary Materials

The following supporting information can be downloaded at: https://www.mdpi.com/article/10.3390/cancers15245806/s1, Table S1: Search Strategy Algorithms in the three major databases; Figure S1: Funnel Plots for pooled estimate of the local tumor progression rates between thermal ablation margins of: (A) <5 mm vs. ≥5 mm (B) <5 mm vs. ≥5 mm using 3D confirmation, (C) <5 mm vs. ≥5 mm stratified by *KRAS* mutation status, (D) <10 mm vs. ≥10 mm and (E) ≥5 mm & <10 mm vs. ≥10 mm.
