# Supplementary material for: The Importance of Optimal Thermal Ablation Margins in Colorectal Liver Metastases: A Systematic Review and Meta-Analysis of 21 Studies
Source: Cancers (Basel). 2023 Dec 12;15(24):5806. doi: 10.3390/cancers15245806 (PMC10741591; doi:10.3390/cancers15245806)
Supplement: Supplementary file 1 [file cancers-15-05806-s001.zip › cancers-2740512-supplementary.pdf]

Database Screened and Results	Search Terms	Limits Used
**MEDLINE**<br><br>(150 Results)	*(("colorectal"[All Fields] AND ("liver"[MeSH Terms] OR "liver"[All Fields] OR "livers"[All Fields] OR "liver s"[All Fields]) AND ("metastasi"[All Fields] OR "neoplasm metastasis"[MeSH Terms] OR ("neoplasm"[All Fields] AND "metastasis"[All Fields]) OR "neoplasm metastasis"[All Fields] OR "metastasis"[All Fields])) OR ("colorectal"[All Fields] AND ("liver"[MeSH Terms] OR "liver"[All Fields] OR "livers"[All Fields] OR "liver s"[All Fields]) AND "neoplas*"[All Fields]) OR ("colorectal"[All Fields] AND ("liver"[MeSH Terms] OR "liver"[All Fields] OR "livers"[All Fields] OR "liver s"[All Fields]) AND "canc*"[All Fields]) OR ("colorectal"[All Fields] AND ("liver neoplasms"[MeSH Terms] OR ("liver"[All Fields] AND "neoplasms"[All Fields]) OR "liver neoplasms"[All Fields] OR ("liver"[All Fields] AND "tumor"[All Fields]) OR "liver tumor"[All Fields]))) AND ((("thermal"[All Fields] OR "thermalization"[All Fields] OR "thermalize"[All Fields] OR "thermalized"[All Fields] OR "thermalizes"[All Fields] OR "thermalizing"[All Fields] OR "thermally"[All Fields] OR "thermals"[All Fields]) AND ("ablate"[All Fields] OR "ablated"[All Fields] OR "ablates"[All Fields] OR "ablating"[All Fields] OR "ablation"[All Fields] OR "ablational"[All Fields] OR "ablations"[All Fields])) OR ("thermoablation"[All Fields] OR "thermoablations"[All Fields] OR "thermoablative"[All Fields]) OR (("microwavable"[All Fields] OR "microwaveable"[All Fields] OR "microwaved"[All Fields] OR "microwaves"[MeSH Terms] OR "microwaves"[All Fields] OR "microwave"[All Fields] OR "microwaving"[All Fields]) AND ("ablate"[All Fields] OR "ablated"[All Fields] OR "ablates"[All Fields] OR "ablating"[All Fields] OR "ablation"[All Fields] OR "ablational"[All Fields] OR "ablations"[All Fields])) OR ("radiofrequency ablation"[MeSH Terms] OR ("radiofrequency"[All Fields] AND "ablation"[All Fields]) OR "radiofrequency ablation"[All Fields])) AND ("margin"[All Fields] OR "margin s"[All Fields] OR "marginal"[All Fields] OR "marginals"[All Fields] OR "margined"[All Fields] OR "margins"[All Fields])*	English Language
**EMBASE (*Scopus*)**<br><br>(225 Results)	*TITLE-ABS-KEY (((colorectal AND (liver OR liver OR livers OR "liver s") AND (metastasi OR "neoplasm metastasis" OR (neoplasm AND metastasis) OR "neoplasm metastasis" OR metastasis)) OR (colorectal AND (liver OR liver OR livers OR "liver s") AND neoplas*) OR (colorectal AND (liver OR liver OR livers OR "liver s") AND canc*) OR (colorectal AND ("liver neoplasms" OR (liver AND neoplasms) OR "liver neoplasms" OR (liver AND tumor) OR "liver tumor"))) AND (((thermal OR thermalization OR thermalize OR thermalized OR thermalizes OR thermalizing OR thermally OR thermals) AND (ablate OR ablated OR ablates OR ablating OR ablation OR ablational OR ablations)) OR (thermoablation OR thermoablations OR thermoablative) OR ((microwavable OR microwaveable OR microwaved OR microwaves OR microwaves OR microwave OR microwaving) AND (ablate OR ablated OR ablates OR ablating OR ablation OR ablational OR ablations)) OR ("radiofrequency ablation" OR (radiofrequency AND ablation) OR "radiofrequency ablation")) AND (margin OR "margin s" OR marginal OR marginals OR margined OR margins))*	English Language
**Cochrane Library**<br><br>(23 Results)	*((colorectal AND ([mh liver] OR liver OR livers OR "liver s") AND (metastasi OR [mh "neoplasm metastasis"] OR (neoplasm AND metastasis) OR "neoplasm metastasis" OR metastasis)) OR (colorectal AND ([mh liver] OR liver OR livers OR "liver s") AND neoplas*) OR (colorectal AND ([mh liver] OR liver OR livers OR "liver s") AND canc*) OR (colorectal AND ([mh "liver neoplasms"] OR (liver AND neoplasms) OR "liver neoplasms" OR (liver AND tumor) OR "liver tumor"))) AND (((thermal OR thermalization OR thermalize OR thermalized OR thermalizes OR thermalizing OR thermally OR thermals) AND (ablate OR ablated OR ablates OR ablating OR ablation OR ablational OR ablations)) OR (thermoablation OR thermoablations OR thermoablative) OR ((microwavable OR microwaveable OR microwaved OR [mh microwaves] OR microwaves OR microwave OR microwaving) AND (ablate OR ablated OR*	English Language

	ablates OR ablating OR ablation OR ablational OR ablations)) OR ([mh "radiofrequency ablation"] OR (radiofrequency AND ablation) OR "radiofrequency ablation")) AND (margin OR "margin s" OR marginal OR marginals OR margined OR margins)	

Supplementary Table 1. Search Strategy Algorithms in the three major databases.

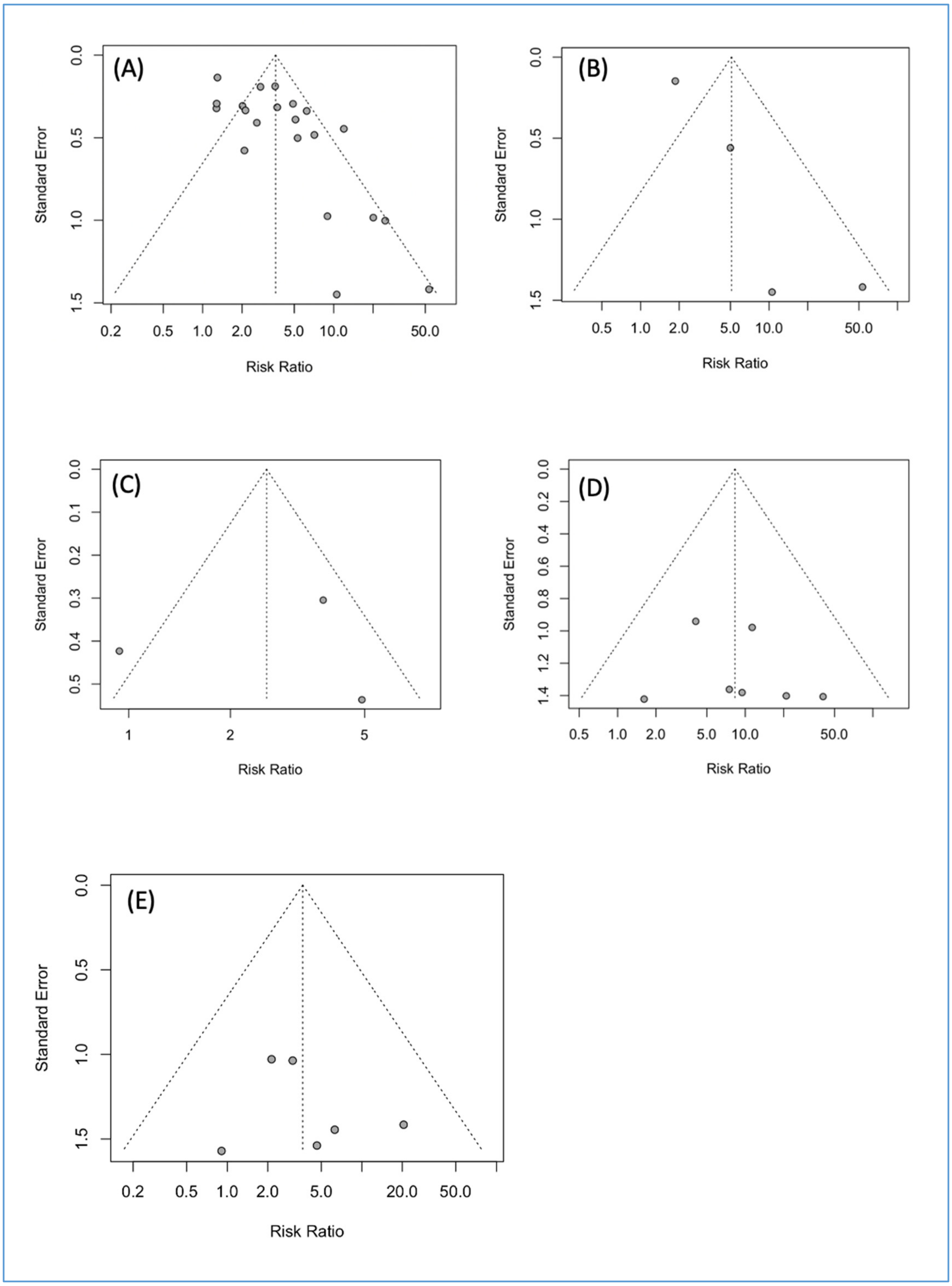

Supplementary Figure 1: Funnel Plots for pooled estimate of the local tumor progression rates between thermal ablation margins of: (A) <5mm vs ⩾5mm (B) <5mm vs ⩾5mm using 3D confirmation, (C) <5mm vs ⩾5mm stratified by *KRAS* mutation status, (D) <10mm vs ⩾10mm and (E) ⩾5mm & <10mm vs ⩾10mm.
